# Supplementary material for: A message passing framework for precise cell state identification with scClassify2
Source: Genome Biol. 2025 Aug 19;26:252. doi: 10.1186/s13059-025-03722-3 (PMC12362893; doi:10.1186/s13059-025-03722-3)
Supplement: Supplementary file 1 — Additional file 1: Fig. S1. The comparison among several graph neural network architectures. The overall prediction accuracy of MPNN and graph transformer is approximately equal, outperforming GCN and GAT. However, the graph transformer consumes significantly more training time compared with MPNN. [file 13059_2025_3722_MOESM1_ESM.docx]

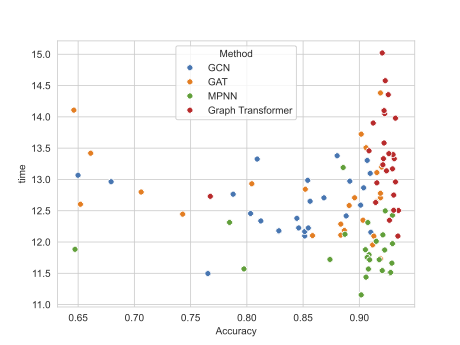


**Fig. S1.** The comparison among several graph neural network architectures. The overall prediction accuracy of MPNN and graph transformer is approximately equal, outperforming GCN and GAT. However, the graph transformer consumes significantly more training time compared with MPNN.
