# Supplementary material for: A message passing framework for precise cell state identification with scClassify2
Source: Genome Biol. 2025 Aug 19;26:252. doi: 10.1186/s13059-025-03722-3 (PMC12362893; doi:10.1186/s13059-025-03722-3)
Supplement: Supplementary file 2 — Additional file 2: Fig. S2. The comprehensive hyperparameter search was conducted for scClassify2. Network hyperparameters of 12 aspects from 3 domains were involved to optimise its performance and robustness for real-world applications. [file 13059_2025_3722_MOESM2_ESM.docx]

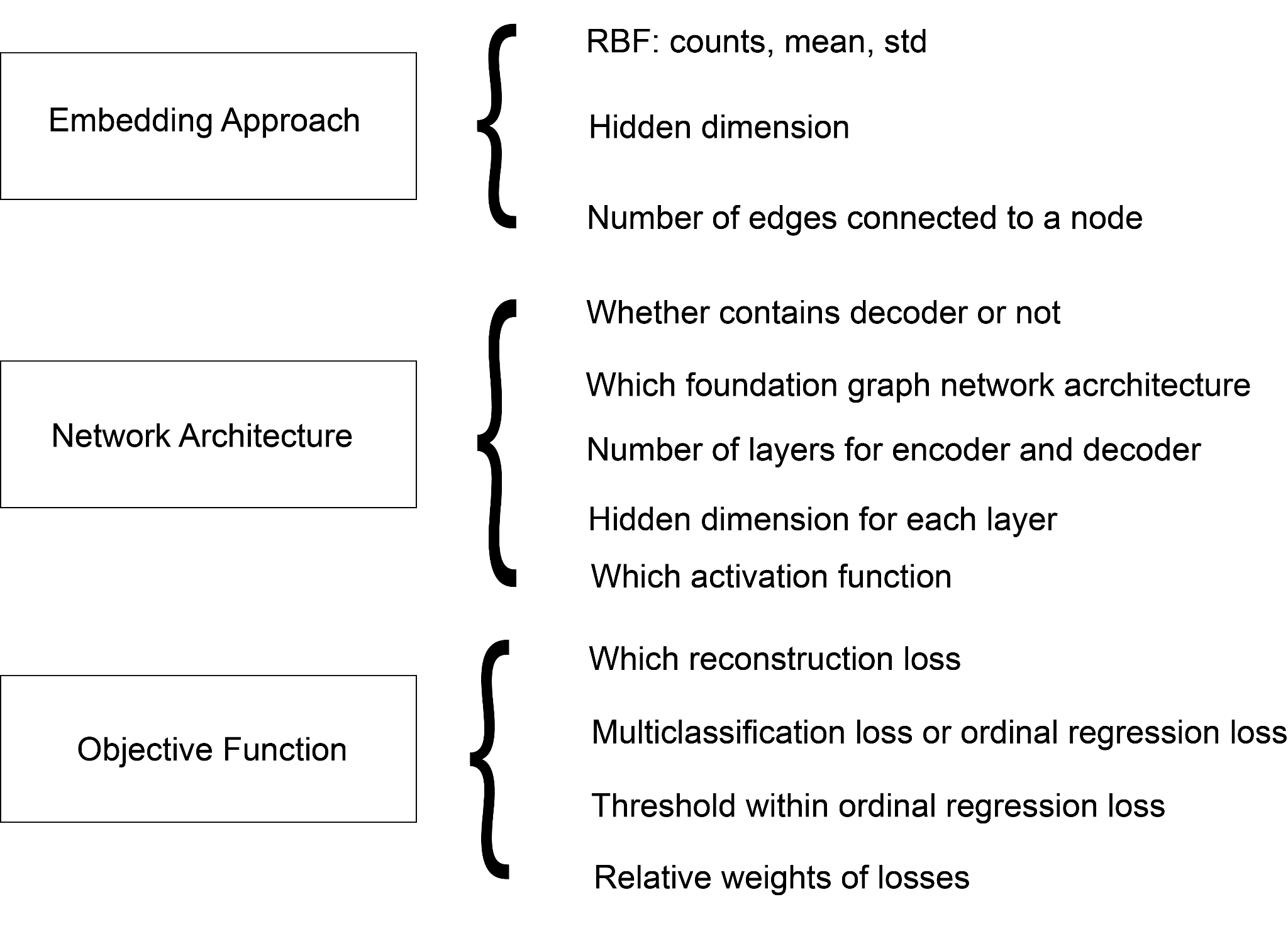


##### **Fig. S2.** The comprehensive hyperparameter search was conducted for scClassify2. Network hyperparameters of 12 aspects from 3 domains were involved to optimise its performance and robustness for real-world applications.
