# Supplementary figures and images for: A message passing framework for precise cell state identification with scClassify2
Source: Genome Biol. 2025 Aug 19;26:252. doi: 10.1186/s13059-025-03722-3 (PMC12362893; doi:10.1186/s13059-025-03722-3)

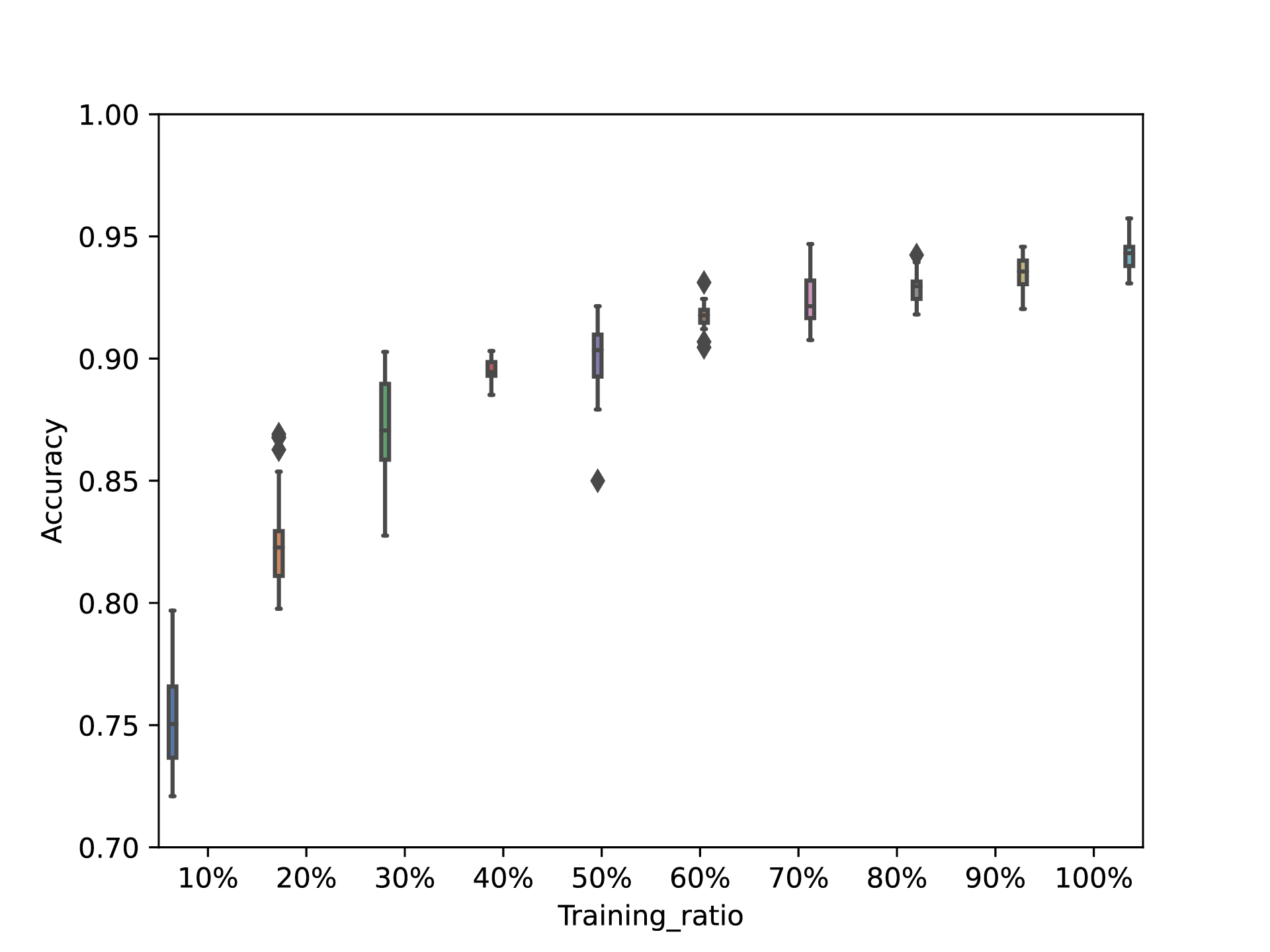


##### **Fig. S3.** The stability of model performance with respect to changes in the size of the training set.

Supplement: Supplementary file 3 — Additional file 3: Fig. S3. The stability of model performance with respect to changes in the size of the training set. [file 13059_2025_3722_MOESM3_ESM.docx]
