# Supplementary material for: A message passing framework for precise cell state identification with scClassify2
Source: Genome Biol. 2025 Aug 19;26:252. doi: 10.1186/s13059-025-03722-3 (PMC12362893; doi:10.1186/s13059-025-03722-3)
Supplement: Supplementary file 4 — Additional file 4: Table S1. Details of eight scRNA-seq datasets of sequential cell state transformation. The columns #cells, #genes, #states denote the number of cells, the number of genes and the number of cell states respectively. [file 13059_2025_3722_MOESM4_ESM.docx]

##### **Table S1.** Details of eight scRNA-seq datasets of sequential cell state transformation. The columns #cells, #genes, #states denote the number of cells, the number of genes and the number of cell states respectively.

| **Datasets** | **Name of dataset** | **First author or organisation** | **Paper** | **#cells** | **#genes** | **#states** | **Species** | **Data accession** |
| --- | --- | --- | --- | --- | --- | --- | --- | --- |
| 1 | Mouse embryo | Wang et al | Nature Communications 14.1 (2023). | 13377 | 29452 | 6 | Mouse | https://github.com/MarioniLab/EmbryoTimecourse2018 |
| 2 | T-cell development | Fernandez et al | Cell Reports  41.7 (2022). | 5859 | 12868 | 5 | Human | GSE211602 |
| 3 | Human embryos | Petropoulos et al | Cell, 165 (2016). | 1289 | 8772 | 5 | Human | E-MTAB-3929 |
| 4 | Mouse oligodendrocyte | Marques et al | Science,352 (2016). | 3685 | 3534 | 10 | Mouse | GSE75330 |
| 5 | Mouse bone marrow mesenchyme  erythrocyte | MCA | Cell, 172 (2018). | 3105 | 3025 | 3 | Mouse | GSE108097 |
| 6 | Mouse dentate gyrus neurogenesis | Hochgerner et al | Nature neuroscience 21.2 (2018). | 3585 | 2182 | 5 | Mouse | GSE95753 |
| 7 | Mouse kidney brush border | MCA | Cell, 172 (2018). | 1400 | 2951 | 3 | Mouse | GSE108097 |
| 8 | Mouse kidney collecting duct | Park et al | Science, 360 (2018). | 2701 | 2441 | 6 | Mouse | GSE107585 |
