# Supplementary material for: A message passing framework for precise cell state identification with scClassify2
Source: Genome Biol. 2025 Aug 19;26:252. doi: 10.1186/s13059-025-03722-3 (PMC12362893; doi:10.1186/s13059-025-03722-3)
Supplement: Supplementary file 5 — Additional file 5: Supplementary Methods. [file 13059_2025_3722_MOESM5_ESM.docx]

### **Supplementary Methods**

##### **Main parameters of MPNN in scClassify2**

##### Batch size is the number of graph samples processed in one training iteration.

##### Smaller batch size would cause more updates per epoch, which would further accelerate the convergence but have more noises for each update. In contrast, large batch size may generalize better with more stable updates. The convergence might be slow and sometimes it would lead to overfitting. More importantly, it requires more GPU memory. Since our MPNNs operate on gene graphs, batch size is limited by GPU memory together with graph size, so after simple preliminary cross validation (as many following hyperparameters), we choose 16 as our default batch size, i.e., we process 16 cells for one training iteration. It is worth noting that, as many other hyperparameters, scClassify2 provides a convenient interface for users to assign their own preference to batch size setting.

#####

##### Training epoch is the number of times the entire dataset passes through the model during training. Too few epochs would cause underfitting, not enough updates to capture patterns. In contrast, too many epochs would cause overfitting, i.e., the model memorizes training data and loses generalization. We set the default epoch as 28 because our preliminary training showed the model converged at around the 25th epoch. Besides, we also set an early stopping mechanism during training to dynamically prevent overfitting.

##### The number of layers, or the depth of our MPNN is the number of message passing steps. Shallow structure would restrict the model in short-range dependencies, while deep structure allows the model to capture global dependencies but risks over-smoothing, where node features become too similar across the graph. By experience, many graph-based tasks benefit from 2 to 4 layers, as deeper models often don’t significantly improve performance. Here, according to our preliminary experiments, we set it to 3. It must be noted that due to the special message passing mechanism of MPNN, there are 3-layer perceptrons for message computation of both vertices and edges during their respective updating within each message passing step. To explain this more clearly, we provide a detailed illustration of the whole MPNN model architecture adopted by scClassify2 in our github page.

##### The size of layers, or so-called hidden dimension, is the number of neurons per layer, which affects feature transformation capacity. Smaller hidden dimension would be less expressive, but may also miss complex patterns. In contrast, larger layer size would allow the model to capture more detailed relationships, but sometimes may cause overfitting. Again, according to our preliminary experiments, we adopted a (384,128,128) dimension for each message computation and update. For better understanding of this architecture, we provided a detailed illustration of scClassify2’s architecture.
